# Sinomenine Regulates PSMB9 to Mediate Therapeutic Effects in Rheumatoid Arthritis

**DOI:** 10.3390/cells15111005

**Published:** 2026-05-29

**Authors:** Cui Zhang, Chonkit Lio, Nana Li, Yang Yu, Jinfang Luo

**Affiliations:** 1 Department of Basic Medicine, Department of Pharmacy, Key Laboratory on the Property & Effect of Chinese Medicine (Ethnic Medicine), Guizhou Genuine Herbs Center of Consistency of Utility, Guizhou Key Laboratory of Miao Medicine, Guizhou University of Traditional Chinese Medicine, Guiyang 550025, China; 18419261387@163.com (C.Z.); 18085483692@163.com (N.L.); yu365589343@163.com (Y.Y.); 2 Faculty of Chinese Medicine, Macau University of Science and Technology, Avenida Wailong, Taipa, Macao, China; 3 State Key Laboratory of Quality Research in Chinese Medicine, Macau University of Science and Technology, Avenida Wailong, Taipa, Macao, China

**Keywords:** rheumatoid arthritis, WGCNA, PSMB9, LMP2

## Abstract

**Highlights:**

**What are the main findings?**

- PSMB9 was identified as a hub gene in rheumatoid arthritis
- Sinomenine may interact with PSMB9 and suppresses its expression, thereby reducing inflammatory responses in macrophages.

**What are the implications of the main findings?**

- PSMB9 may have potential relevance as a biomarker in rheumatoid arthritis.
- Targeting PSMB9 may provide a novel mechanistic basis and offers a new strategy for rheumatoid arthritis treatment.

**Abstract:**

Rheumatoid arthritis (RA) is a systemic immune-related disease characterized by chronic synovial inflammation and progressive joint destruction. However, the molecular mechanisms and diagnostic biomarkers underlying RA remain unclear. In this study, we aimed to identify potential biomarkers for clinical diagnosis of RA and to investigate their association with immune infiltration. By integrating differentially expressed genes analysis (DEGs) and weighted gene co-expression network analysis (WGCNA), we identified PSMB9 as a hub gene associated with RA. Gene set enrichment analysis (GSEA) and immune infiltration analysis revealed a strong association between RA and macrophage infiltration. Single-cell RNA sequencing datasets also suggest that PSMB9 is not only highly expressed in macrophage but is also present in synovial cells. We employed cellular thermal shift assay (CETSA) combined with Western blot to validate the interaction between sinomenine (SIN) and the target protein. CETSA results demonstrated that, compared with the control group, SIN increased the thermal stability of PSMB9, suggesting direct binding between the two. Western blot experiments further confirmed that PSMB9 protein expression was significantly downregulated following SIN treatment. PSMB9 may serve as potential diagnostic biomarker and therapeutic targets for RA. Moreover, our data suggest SIN may exert anti-inflammatory effects through regulation of PSMB9. This study also provides an additional insight into the underlying mechanisms involved in the progression of RA and discover a new molecular target for SIN.

## 1. Introduction

Rheumatoid arthritis (RA) is a chronic disorder for which there is no known cure [1]. Its typical pathological changes include long-term inflammation of synovial tissue and progressive damage of articular cartilage [2]. Therefore, it has a high disability rate, which affects the quality of life and life expectancy of patients [3]. According to statistics, RA patients account for about 1% of the global population, and the peak age of onset is between 40 and 50 years old, especially in women [4]. Non-steroidal anti-inflammatory drugs (NSAIDs) [5], glucocorticoids [6], and disease-modifying anti-rheumatic drugs (DMARDs) [7] are commonly used in cases of RA. NSAIDs primarily alleviate acute inflammatory responses, thereby relieving pain and improving function [8,9]. However, NSAIDs do not alter the disease procession or prevent joint destruction [10], and their long-term use may lead to gastrointestinal bleeding [11], renal dysfunction [12], and an increased risk of cardiovascular events [13]. Previous studies have confirmed that glucocorticoids exert effects such as symptom relief, physical function improvement, and quality-of-life enhancement [14], yet they fail to halt or delay bone destruction and may increase the risk of various complications including infection, cardio-cerebrovascular diseases, and osteoporosis [15,16]. DMARDs, an immunosuppressive agents, can ameliorate some symptoms of RA through immunomodulation [17]. DMARDs can effectively slow disease progression and reduce joint damage through immunomodulation; however, they have no obvious pain relief effects [18]. Among them, conventional synthetic DMARDs are associated with adverse reactions including gastrointestinal intolerance, nephrotoxicity [19], and hepatotoxicity [20]; biologic DMARDs may increase the risk of malignancy [21] and cardiovascular events [22]; targeted synthetic DMARDs carry risks such as increased venous thromboembolism [23], malignancy [24], and limitations in clinical application [23].

In recent years, naturally derived active compounds have attracted extensive attention in RA treatment research owing to their advantages including multi-target regulation, low toxicity and side effects, and wide availability [25]. Among them, sinomenine (SIN), an effective monomeric alkaloid extracted from *Sinomenium acutum* of the Menispermaceae family, exhibits significant anti-inflammatory, immunomodulatory, analgesic, and bone-protective effects [26]. Clinical randomized controlled trials have demonstrated that the efficacy of SIN monotherapy in RA is comparable to that of methotrexate (MTX), with an ACR50 response rate of 52.63% after 24 weeks of treatment. Moreover, the incidence of liver injury is significantly lower in the SIN group than in the MTX group, and gastrointestinal adverse reactions are also markedly reduced [27,28]. In addition, the combination of SIN and MTX not only enhances therapeutic efficacy but also mitigates MTX-related hepatotoxicity and gastrointestinal side effects, demonstrating an ideal efficacy–safety balance [28]. These properties render SIN a highly promising candidate agent in combination therapeutic strategies for RA.

Proteasome subunit beta type-9 (PSMB9), also known as low molecular mass protein 2 (LMP2), is a key catalytic subunit of the 20S core particle of the immunoproteasome [29]. PSMB9 plays a crucial role in intracellular protein degradation, cleaving proteins into small peptide fragments that are transported to the endoplasmic reticulum via TAP1 and loaded onto MHC class I molecules for antigen presentation [30]. PSMB9 is transcriptionally upregulated by interferon-γ (IFN-γ), highlighting its important role in the immune response [31]. Dysregulation of the immunoproteasome is implicated in multiple diseases, including autoimmune diseases [32], cerebral ischemia [33], and atherosclerosis [34]. As an autoimmune disorder, RA may benefit from novel therapeutic strategies targeting PSMB9.

Based on the above background, this study aims to elucidate the pivotal role of PSMB9 in the pathophysiological process of RA and systematically evaluate the interaction between SIN and the PSMB9 protein. Through integrated bioinformatics analysis, molecular docking, and in vitro cellular functional assays, we reveal for the first time a novel molecular mechanism by which SIN modulates immunoproteasome activity via direct binding to PSMB9, thereby intervening in the immune-inflammatory response in RA. This finding not only expands the understanding of the multi-target pharmacological effects of SIN but also provides an important theoretical basis and experimental foundation for the development of precision therapeutic strategies targeting PSMB9 for RA.

## 2. Materials and Methods

### 2.1. Dataset Collection and Preprocessing

We obtained 4 gene-expression datasets (GSE276772, GSE285977, GSE55457, and GSE77298) from the GEO database. GSE55457 and GSE77298 consist of synovial tissue samples from patients with rheumatoid arthritis (RA) and healthy controls, which were used to identify disease-associated transcriptional changes. In contrast, GSE276772 and GSE285977 are gene perturbation datasets or cytokine stimulation, which were used to explore gene regulatory mechanisms.

All GEO datasets were processed and analyzed independently. To avoid cross-platform batch effects, expression matrices were not merged. Differentially expressed genes were identified within each cohort, and only the gene-level results were compared across datasets for consensus analysis. Missing values were handled using the impute (v1.76.0) R package to ensure data Integrity. Because we obtained both microarray and RNA-seq platforms datasets from GEO database, we processed them separately.

For microarray datasets, CEL files were imported into R using affy package (v1.80.0) and preprocessed with robust multi-array average (RMA), which includes background correction, quantile normalization, and probe-set summarization to log2-scaled expression values (affy::rma). The normalized expression matrix was extracted using exprs for downstream analyses. Probe sets were annotated using platform-specific annotation packages, and probe-to-gene mapping was performed to obtain gene symbols and Entrez IDs prior to differential analysis.

The RNA-seq dataset was processed using DESeq2 (v1.42.1) R package following standard workflows. To identify transcriptional differences between RA and control synovial tissues, we applied limma (v3.58.1) R package for the microarray datasets and DESeq2 for the RNA-seq dataset. Those genes with *p* < 0.05 and |log_2_FC| > 0.5 were believed differentially expressed. All visualization of the DEG results was performed using ggplot2 (v4.0.1) R package.

### 2.2. Immune Infiltration Analyses

We estimated immune cell infiltration using the xCell (v1.1.0) algorithm implemented in R (v.4.3.2) Expression matrices from the GSE55457 and GSE77298 datasets were normalized and log_2_ transformed before further analysis. The result of xCell infiltration (enrichment) scores were scaled to allow cross-sample comparison. Differences in immune infiltration between the RA and control groups were evaluated using the Wilcoxon rank-sum test. All figures were generated in R using the ggplot2 package.

### 2.3. Screening for Candidate Gene Modules Using WGCNA

Weighted gene co-expression network analysis (WGCNA) was performed on the full gene-expression matrix without removing genes except that extremely lowly expression. The WGCNA (v.1.73) R package was used to construct a signed co-expression network. We selected the lowest soft-thresholding power (β) that satisfied the scale-free topology criterion. A topological overlap matrix (TOM) was calculated, and hierarchical clustering of the TOM-based dissimilarity measure was used to detect gene modules. Module boundaries were refined using the dynamicTreeCut algorithm. Module–trait associations were assessed using Pearson correlation (*p* < 0.05). Hub genes within the relevant module were identified based on high intramodular connectivity and visualized in R using ggplot2.

### 2.4. Function Enrichment Determination

We performed Gene Ontology (GO) and Kyoto Encyclopedia of Genes and Genomes (KEGG) enrichment analyses using the clusterProfiler (v4.11.0) R package. Enrichment analyses were performed separately for human (org.Hs.eg.db) and mouse (org.Mm.eg.db) gene annotations. DEGs were first converted to Entrez IDs using the bitr function prior to enrichment. GO enrichment analysis was carried out for biological process (BP), cellular component (CC), and molecular function (MF) categories, with a significance threshold of adjusted *p*-value < 0.05. KEGG pathway analysis was performed using the enrichKEGG function, and pathways with *p* < 0.05 were selected for the enrichment result visualization. We visualized enrichment results using the enrichplot (v1.22.0) and ggplot2 packages, and bubble plots for both GO and KEGG outputs were generated.

We then performed gene set enrichment analysis (GSEA) using the clusterProfiler package. Gene sets were retrieved from the Molecular Signatures Database (MSigDB) via the msigdbr (v25.1.1) R package. DEGs were ranked by log_2_ fold-change before running the GSEA function. Following standard recommendations, we set the minimum and maximum gene set sizes to 10 and 500, respectively.

### 2.5. Protein–Protein Interaction (PPI) Network Analysis

To identify high-confidence protein–protein interactions and to explore the functional connectivity of the target gene, we used the STRING database (https://string-db.org/) with a minimum interaction confidence score of 0.95 to generate PPI networks. Analyses were performed using either a single input gene or a three-gene set, corresponding to the networks shown in the results. Network visualization was carried out directly on the STRING web platform.

### 2.6. Single-Cell RNA-Seq Data Processing

The single-cell RNA sequencing data used in this study were obtained from the GEO database (GSE109449). According to the original dataset description, synovial tissue samples were collected from joint synovium of patients with RA and osteoarthritis (OA) individuals. Relevant clinical and sample source information was incorporated to ensure clarity in data interpretation. The analyses of single-cell RNA-seq dataset were performed starting from a gene-by-cell TSV expression matrix that has been already finished quality control such as cell calling and initial alignment/UMI processing was downloaded from NCBI/GEO database. Raw count matrices (gene × cell) from four donors (OA4, OA5, RA8, RA9) comprising 396 synovial fibroblast cells were imported into R and processed as follows. Genes with all-zero expression across cells were removed. Library sizes were computed for each cell and counts were normalized using the counts-per-million (CPM) method:

$${\mathrm{CPM}}_{\mathrm{ij}}\;=\;\frac{x_{\mathrm{ij}}}{\sum_{j}x_{\mathrm{ij}}}\text{×}10^{6}$$

Log-transformed CPM values were obtained as:

$$\log_{2}\left(\mathrm{CPM}+1\right)$$

These values were used for all downstream analyses. Metadata were constructed based on cell IDs, assigning each cell to its donor and disease group (OA or RA).

### 2.7. GSVA Pathway Enrichment Analysis

We retrieved Hallmark gene sets using the msigdbr (v25.1.1) R package and computed pathway activity scores using the GSVA package (v1.50.5). We then focused on immune-related Hallmark pathways to compare pathway activity between OA and RA pseudo-bulk fibroblast samples and visualized pathway enrichment using ggplot2.

### 2.8. Correlation Analysis Between PSMB9 and Immune Pathways

We paired donor-level PSMB9 expression (logCPM) with corresponding GSVA pathway scores and calculated Spearman correlation coefficients to quantify the association between PSMB9 and immune pathway activation. We visualized each relationship using scatterplots with fitted regression lines generated in ggplot2.

### 2.9. Molecular Docking

The PSMB9 crystal structure (PDB ID: 6avo) was obtained from the PDB database and saved in PDF format. The 3D structure of SIN was downloaded from the PubChem database and saved in SDF format. The downloaded small molecules and target proteins were imported into the CB-DOCK2 platform https://cadd.labshare.cn/cb-dock2/php/index.php (accessed on 20 August 2025) for structure-based molecular docking. The docking pose exhibiting the lowest Vina score was selected as optimal. Based on the molecular docking analysis, the binding affinity score serves as a candidate indicator for evaluating the interaction between the ligand and the target protein. A binding affinity lower than −4.25 kcal/mol suggests that the compound has the potential to bind to the target, scores below −5.0 kcal/mol denote robust, favorable binding, and a score < −7.0 kcal/mol indicates that the two have strong binding activity.

### 2.10. Experimental Validation

#### 2.10.1. Main Reagents and Instruments

The following reagents and instruments were used in this study: SIN, purity > 99%, was obtained from Chengdu Ruifen Sidedan Biotechnology Co., Ltd., Chengdu, China. Lipopolysaccharide (LPS) and dexamethasone (DEX) were sourced from Sigma-Aldrich (St. Louis, MO, USA). The RAW264.7 cell line was acquired from the China Center for Type Culture Collection at Wuhan University. The CCK8 assay kit was purchased from Biyuntian Biotechnology Co., Ltd., Shanghai, China. Primary antibodies included Rabbit polyclonal PSMB9 antibody (Wuhan Sanying Biotechnology Co., Ltd., Wuhan, China) and Mouse monoclonal β-actin antibody (Wuhan Sanying Biotechnology Co., Ltd., Wuhan, China). ELISA kits for detecting TNF-α and IL-6 were supplied by Hangzhou Youke Life Science and Technology Co., Ltd., Hangzhou, China. The secondary antibody (anti-rabbit or anti-mouse) was purchased from Beijing Solarbio Technology Co., Ltd., Beijing, China. Ultrasensitive multifunctional chemiluminescence imaging system (Bio-Rad, Hercules, CA, USA; Model: ChemiDoc^TM^). electrophoresis and electrotransfer apparatus (Beijing Hongtao Jiye, Beijing, China; Model: HT-Zy02).

#### 2.10.2. Cell Culture and Processing

RAW264.7 cells were cultured in complete DMEM medium supplemented with 10% fetal bovine serum and maintained in a humidified atmosphere of 5% CO_2_, 37 °C constant temperature incubator. When the cells grew to 80% density, they were passaged once for about 18–24 h. After three generations, the cells could be used in the experiment until they were stable.

#### 2.10.3. Cell Viability Assay

CCK8 kit was used to detect cell activity. 1.8 × 10^5^/mL cells were inoculated into 96-well plates (100 μL per well), and the control group and blank group were set up at the same time, and incubated overnight at 37 °C. Subsequently, the cells were cultured in a drug-containing medium containing SIN concentration gradients (150, 300, 600, 1200, and 2400 μM) for 18 h. After treatment, 10 μL of CCK-8 solution was added to each well, followed by incubation at 37 °C for 45 min to 1 h. Absorbance at 450 nm was then measured using a microplate reader. The relative cell viability was calculated with the following formula: Relative viability (%) = [(ODtreatment − ODblank)/(ODcontrol − ODblank)] × 100%.

#### 2.10.4. ELISA Detection

TNF-α and IL-6 were determined by ELISA. RAW264.7 cells (3 × 10^5^ cells/mL) were plated in 6-well plates and maintained overnight at 37 °C. Following 1 h exposure to SIN or DEX, cultures were challenged with LPS (1 μg/mL) for 18 h. Supernatants were harvested, clarified (1200 rpm, 10 min), and processed per the kit instructions; absorbances were recorded at 450 nm with 570 nm background correction.

#### 2.10.5. Western Blot Detection

RAW264.7 cells were seeded into 6-well plates at a density of 2 mL per well and incubated for 18 h at 37 °C under 5% CO_2_ in a humidified atmosphere. According to the low, medium, and high concentration settings of SIN drugs, different volumes of drug solution were added to each well so that the final concentration was the expected concentration, and the final concentration of DEX group was 1 μM. Following a 1-h treatment period, LPS solution was added to each well to achieve a final concentration of 1 μg/mL. The normal group and the model group were set up, and the culture was continued for 18 h. The well plate was taken out, washed with PBS, and the cells were lysed with RIPA lysate. The protein samples were resolved on a 10% SDS-polyacrylamide gel and transferred onto a PVDF membrane. The membrane was then blocked at room temperature for 45 min, followed by overnight incubation at 4 °C with primary antibodies against PSMB9 (1:1000) and β-actin (1:3000). The membranes were washed four times with 1 × TBST and then incubated for 1 h at room temperature with corresponding HRP-conjugated secondary antibodies (1:3000). After another four washes with TBST, protein bands were visualized using a chemiluminescence imaging system and quantified by analyzing the grayscale values with ImageJ software (v1.53a).

#### 2.10.6. CETSA Validation

RAW264.7 cells were seeded at a density of 2 × 10^6^ cells/well in 6-well plates and incubated for 24 h at 37 °C under 5% CO_2_ in a humidified atmosphere. Cells were then treated with either DMSO (vehicle control) or SIN (600 μM) for 19 h. After treatment, cells were washed twice with ice-cold PBS, harvested by scraping, and centrifuged at 1000 rpm for 5 min. Cell pallets were resuspended in PBS to a density of 5 × 10^5^ cells/100 μL and aliquoted into PCR tubes. Sample were subjected to a temperature gradients (53, 63, 73, 83, 90, and 98 °C) for 3 min using a dry bath, then immediately freezing at −80 °C for 10 min. Freeze–thaw cycles were repeated 3 times. Then, cells were lysed in RIPA buffer on ice for 30 min and centrifuged at 12,000 rpm at 4 °C for 20 min. The supernatant was collected for protein quantification using a BCA assay. Sample were then mixed with loading buffer, boiled at 95 °C for 10 min, and analyzed by Western blot. Results were analyzed using ImageJ software for grayscale quantification.

#### 2.10.7. Statistical Analysis

Statistical analysis and data visualization were conducted using GraphPad Prism 8.0.2 One-way ANOVA with Tukey’s post hoc test was applied for multi-group comparisons. Results are expressed as mean ± standard deviation, with a *p*-value < 0.05 considered statistically significant.

## 3. Results

### 3.1. Comparative Analysis of DEGs Across Multiple Datasets and Functional Enrichment

**Figure 1:**
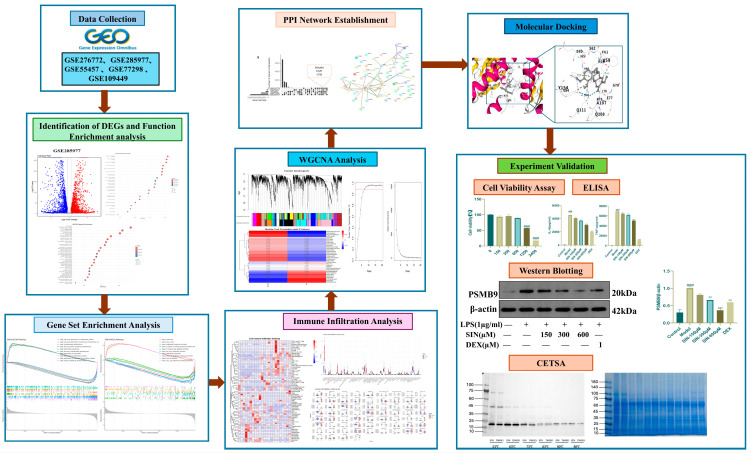
Schematic representation of the analysis workflow. This figure illustrates the overall research workflow, including data preprocessing, feature selection, differential expression analysis, functional enrichment (KEGG/GO), network analysis (PPI/WGCNA) and UpSetR analysis. Arrows indicate the sequential steps of the analysis pipeline.

**Figure 2:**
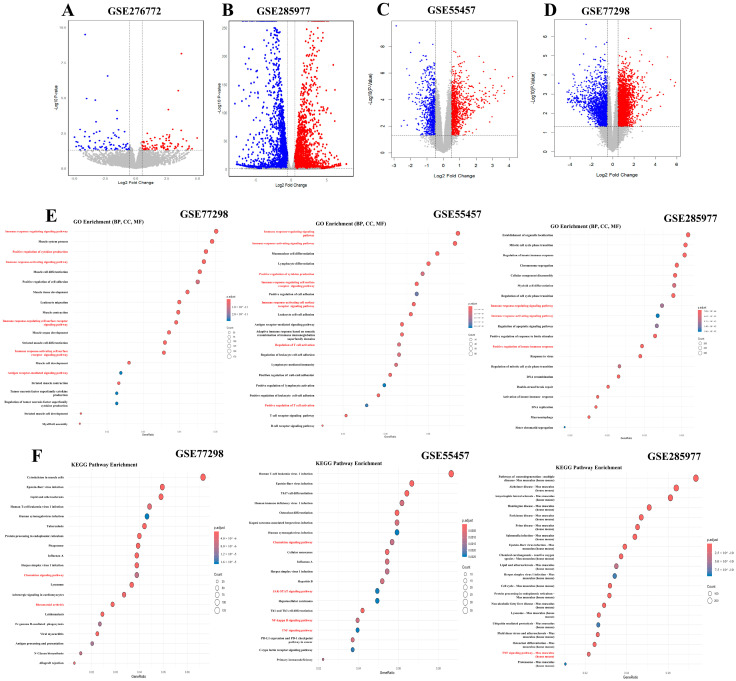
Differential gene expression and functional enrichment analysis. (**A**–**D**) Volcano plot showing differentially expressed genes (DEGs) identified by RNA-seq analysis. Upregulated genes are shown in red and downregulated genes in blue, with significance thresholds indicated by dashed lines. (**E**) Gene Ontology (GO) enrichment analysis of DEGs, including biological process (BP), cellular component (CC), and molecular function (MF). Bubble size indicates gene count, and color represents statistical significance. (**F**) KEGG pathway enrichment analysis of DEGs. Bubble size reflects the number of enriched genes, and color indicates adjusted *p*-values (FDR). Red text indicates immune- or inflammation-related GO terms/pathways highlighted for emphasis.

The operation flow of this study was illustrated in Figure 1. Across four independent cohorts, each dataset revealed a distinct DEG profile. We identified 174 DEGs, including 90 upregulated and 84 downregulated genes in GSE276772 dataset (Figure 2A). In GSE285977 dataset, 9729 DEGs with 3131 genes upregulated and 6598 genes downregulated were detected under the same condition (Figure 2B). In the GSE55457 dataset, 1910 DEGs were detected, comprising 1168 upregulated and 742 downregulated genes (Figure 2C). Similarly, the GSE77298 dataset yielded 5408 DEGs, including 3632 upregulated and 1776 downregulated genes (Figure 2D).

We next examined whether these genes converged on shared biological processes. To do that, we did GO and KEGG enrichment analyses and the result revealed that the DEGs were predominantly enriched in immune- and inflammation-related pathways (Figure 2E,F). Several of the top enriched terms were closely linked to macrophage biology, including regulation of macrophage activation, negative regulation of IL-10 production, inflammatory response, and bone resorption. In addition, enrichment of the IL-15–mediated signaling pathway suggests potential crosstalk between innate immune cells and inflammatory signaling networks.

Because DEG-based enrichment focuses only on a subset of significantly altered genes, broader pathway-level transcriptional shifts may be underestimated. To address this limitation, we next applied GSEA using the same datasets to find coordinated expression changes across the entire transcriptome.

**Figure 3:**
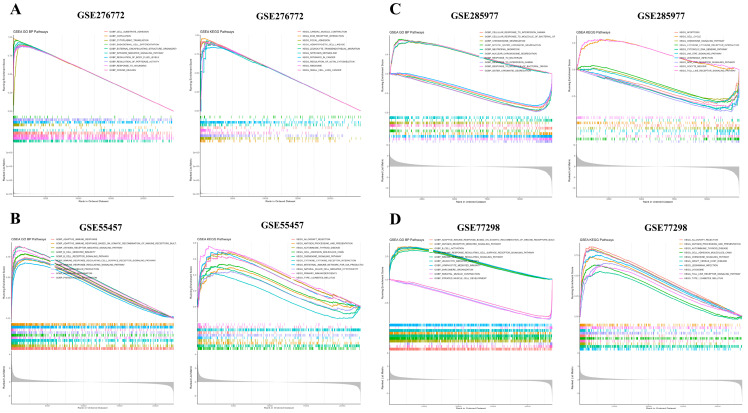
Gene set enrichment analysis (GSEA) of KEGG pathways and Gene Ontology (GO) terms. (**A**–**D**, left panel) The enrichment plot displays the top 10 significantly enriched GO BP pathways ranked by enrichment score. The x-axis represents the ranked gene list, and the y-axis shows the enrichment score across gene ranks. Colored curves indicate different GO terms, with peaks representing the maximum enrichment score. Only pathways meeting the predefined significance threshold (e.g., FDR < 0.05) are displayed. (**A**–**D**, right panel) The enrichment plot displays the top 10 significantly enriched KEGG pathways ranked by enrichment score. The x-axis represents the ranked gene list, and the y-axis shows the enrichment score across gene ranks. Colored curves indicate different KEGG pathways, with peaks representing the maximum enrichment score. Only pathways meeting the predefined significance threshold (e.g., FDR < 0.05) are displayed.

JAK–STAT and Toll-like receptor pathways, both of which are central to innate immune activation and myeloid cell function, were confirmed in GSEA-KEGG. At the same time, GSEA-GO revealed coordinated upregulation of interferon-related responses and NF-κB signaling (Figure 3A,D). Both were not apparent in the DEG-based analysis. Taken together, these results suggest that global transcriptional reprogramming of innate immune pathways—particularly those associated with macrophage activation and inflammatory signaling—may contribute to disease progression.

### 3.2. Infiltration of Immune Cells in RA and Control Synovial Tissues

**Figure 4:**
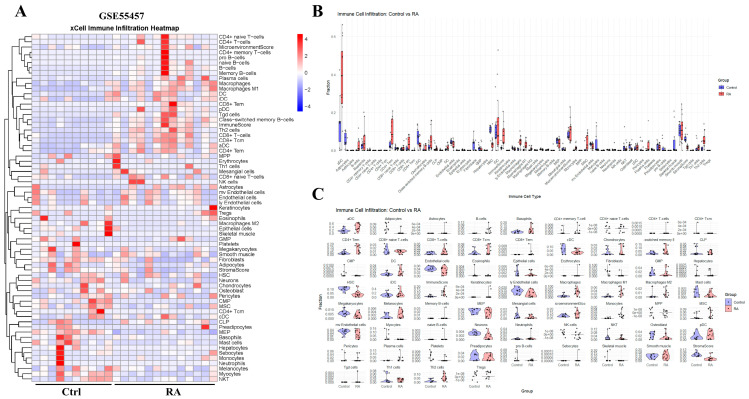
xCell-based immune infiltration analysis in the GSE55457 dataset. (**A**) Heatmap of immune cell composition inferred by xCell across all samples. Rows correspond to immune cell types and columns to individual samples, with color intensity reflecting relative infiltration levels. (**B**) Bar chart summarizing the immune cell infiltration patterns among different sample groups. (**C**) Violin plots depicting group-wise differences in selected immune cell populations, where the distribution width represents the density of infiltration scores.

**Figure 5:**
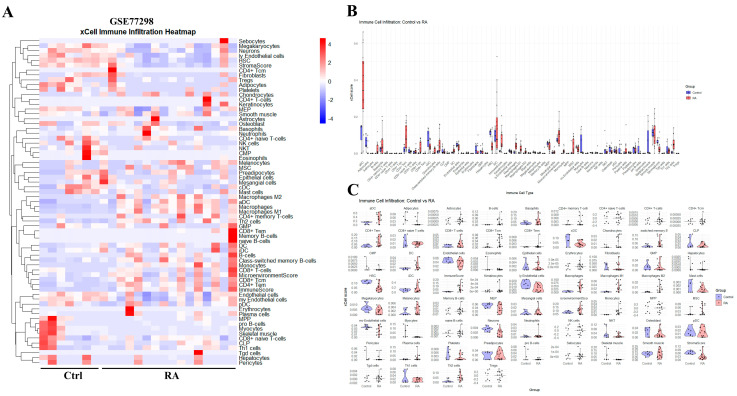
xCell-based immune infiltration analysis in the GSE77298 datasets. (**A**) Heatmap of immune cell composition inferred by xCell across all samples. Rows correspond to immune cell types and columns to individual samples, with color intensity reflecting relative infiltration levels. (**B**) Bar chart summarizing the immune cell infiltration patterns among different sample groups. (**C**) Violin plots depicting group-wise differences in selected immune cell populations, where the distribution width represents the density of infiltration scores.

Based on the diagnostic immune-related pathways identified above, we next quantified immune cell infiltration by comparing normal and RA synovial tissues. As shown in Figure 4, RA synovium exhibited markedly increased enrichment of multiple immune cell populations compared with controls. Macrophages, especially M1 macrophages, were the most prominently enriched in RA samples (Figure 4A–C). To further validate this finding, the same analysis was performed using the GSE77298 dataset. Consistent infiltration patterns were observed, with M1 macrophages, plasma cells and CD8^+^ T cells again showing substantial enrichment in RA synovium (Figure 5A). Violin plot comparisons further demonstrated significantly elevated levels of these immune cell subsets in RA (Figure 5B). Like GSE55457, M1 macrophages showed the most pronounced increase and were selected for subsequent analyses (Figure 5C).

### 3.3. Identification of Critical Genes and Functional Enrichment Analysis of Candidate Genes in Immune-Related Modules

**Figure 6:**
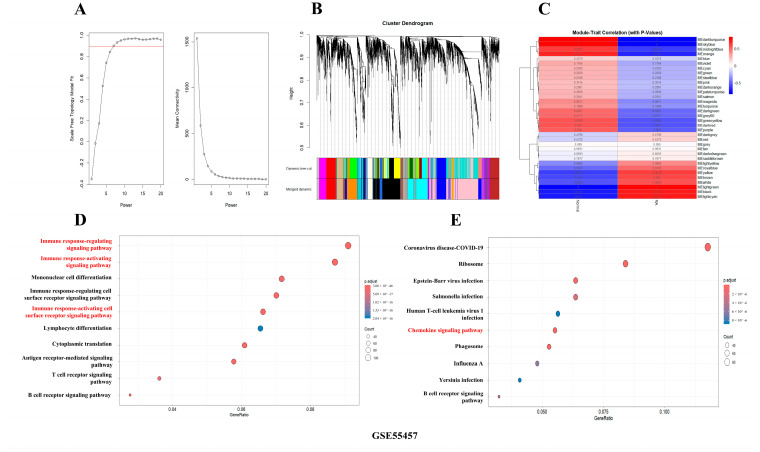
Weighted gene co-expression network analysis (WGCNA) of GSE55457. (**A**) Scale-free topology model fit and mean connectivity plot: The soft-thresholding power selection, where the left panel shows the scale-free topology model fit index as a function of power and the right panel shows mean connectivity. (**B**) Gene clustering dendrogram with module colors: Hierarchical clustering of genes into distinct modules, with colors representing co-expression modules. (**C**) Module–trait relationship heatmap: Correlation between identified modules and sample traits/phenotypes. The color scale represents correlation values, with red indicating positive correlations and blue indicating negative correlations. (**D**) GO enrichment analysis of genes in significant modules: Functional enrichment analysis of genes from key WGCNA modules, identifying significantly GO terms associated with biological processes, molecular functions, and cellular components. (**E**) KEGG pathway analysis of genes in significant modules: Functional enrichment analysis of genes from key WGCNA modules, identifying significantly enriched KEGG pathways. Red text indicates immune- or inflammation-related GO terms/pathways highlighted for emphasis.

To identify gene co-expression patterns associated with RA, we performed a WGCNA based on the GSE55457 synovial transcriptome dataset. The soft-thresholding power β = 7 was selected to achieve scale-free topology (Figure 6A), and the resulting hierarchical clustering grouped genes into multiple distinct modules (Figure 6B). Module–trait correlation analysis revealed that seven modules, including the lightcyan, black, and lightgreen modules, showed strong positive correlations with RA status, whereas four modules exhibited negative correlations (Figure 6C).

We then preformed enrichment analysis of genes in *p* < 0.05 modules chosen from the GSE55457 datasets based on WGCNA to confirm whether immune-related pathway strongly participate in these key modules. We found that those modules are associated with immune, inflammatory response, and cytokines production. Of note, these are associated with the development and progression of RA (Figure 6D,E). Notably, these pathways are highly relevant to the development and progression of RA, as reported in previous studies [34,35,36].

### 3.4. Genes Overlapping Among Four Independent Ra Datasets and the Ra-Associated Wgcna Module

**Figure 7:**
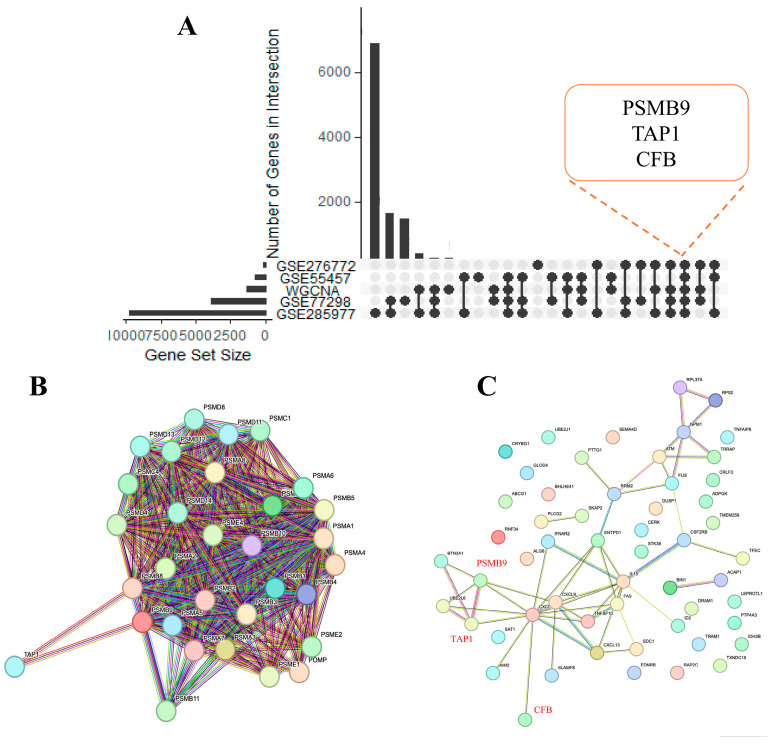
Feature selection and functional analysis of final candidate genes. (**A**) UpSetR plot of overlapping genes: Intersection analysis of key genes identified across multiple selection methods, illustrating shared and unique features. (**B**) PPI network constructed using PSMB9 as the seed gene, highlighting its direct interaction partners. (**C**) PPI network constructed from multiple key genes (PSMB9, TAP1, and CFB), revealing a broader interaction network and potential cooperative relationships.

To identify robust RA-associated genes, we intersected differentially expressed genes (DEGs) from four independent datasets with genes from the RA-associated WGCNA module. This analysis revealed a core set of overlapping genes, including PSMB9, TAP1, and CFB, consistently shared across datasets and network analysis (Figure 7A).

To further characterize the protein interaction landscape of the identified genes, we performed protein–protein interaction (PPI) analysis using two complementary approaches. First, PSMB9 was used as a single seed gene to examine its direct interaction network, revealing a densely connected cluster enriched for proteasome and antigen-processing components (Figure 7B).

To extend this analysis, we next constructed a PPI network using PSMB9, TAP1, and CFB as input genes. This multi-seed approach revealed a broader and more interconnected network, indicating that these RA-associated genes converge on shared immune-related pathways (Figure 7C). Together, these analyses suggest that PSMB9, TAP1, and CFB are embedded within a coordinated protein interaction network rather than acting as isolated factors.

### 3.5. Upregulation of PSMB9 in RA FLS Is Accompanied by Enhanced Interferon and Inflammatory Pathway Activity

**Figure 8:**
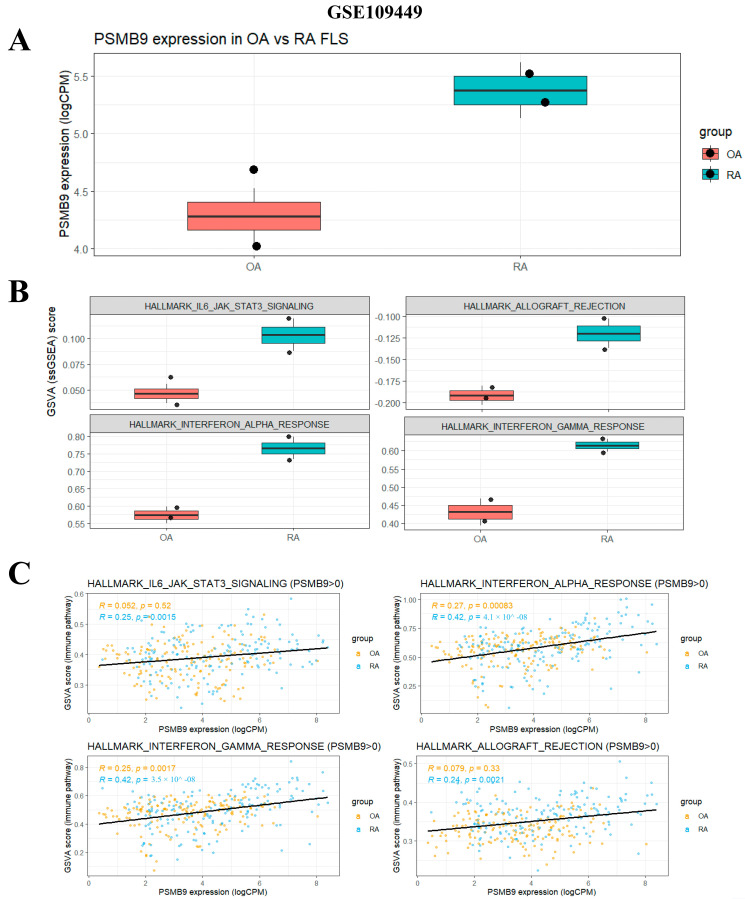
PSMB9 expression and immune pathway activity in OA and RA fibroblast-like synoviocytes. (**A**) Boxplots showing PSMB9 expression (logCPM) in pseudo-bulk OA versus RA FLS. (**B**) GSVA (ssGSEA) scores for four immune-related Hallmark pathways in OA and RA FLS. (**C**) Correlation between PSMB9 expression and pathway GSVA scores across the four donors (OA4, OA5, RA8, RA9).

Since PSMB9 emerged as a key gene in both the WGCNA and protein–protein interaction analyses, we next examined its expression and functional relevance at the single-cell level in fibroblast-like synoviocytes (FLS). Analysis of pseudo-bulk single-cell profiles revealed that PSMB9 expression was significantly higher in RA-derived FLS compared with OA FLS (Figure 8A), indicating disease-associated upregulation.

To determine whether elevated PSMB9 expression reflected a broader inflammatory state, we assessed pathway activity using gene set variation analysis (GSVA). RA FLS displayed significantly increased enrichment of multiple immune and cytokine-related Hallmark pathways, including interferon-α response, interferon-γ response, allograft rejection, and IL6/JAK–STAT3 signaling, all of which showed positive correlations with PSMB9 expression (Figure 8B).

We next explored the cellular heterogeneity underlying this association by analyzing synovial single-cell RNA-seq data. At the single-cell level, PSMB9 expression was enriched in inflammatory FLS subsets, and cells with higher PSMB9 expression exhibited increased activation of interferon-related signaling programs (Figure 8C). Together, these results indicate that PSMB9 marks a pro-inflammatory fibroblast population characterized by coordinated activation of interferon and cytokine signaling pathways, which is selectively enriched in RA synovium.

### 3.6. SIN Binds to and Downregulates PSMB9 to Attenuate Macrophage Inflammation

**Figure 9:**
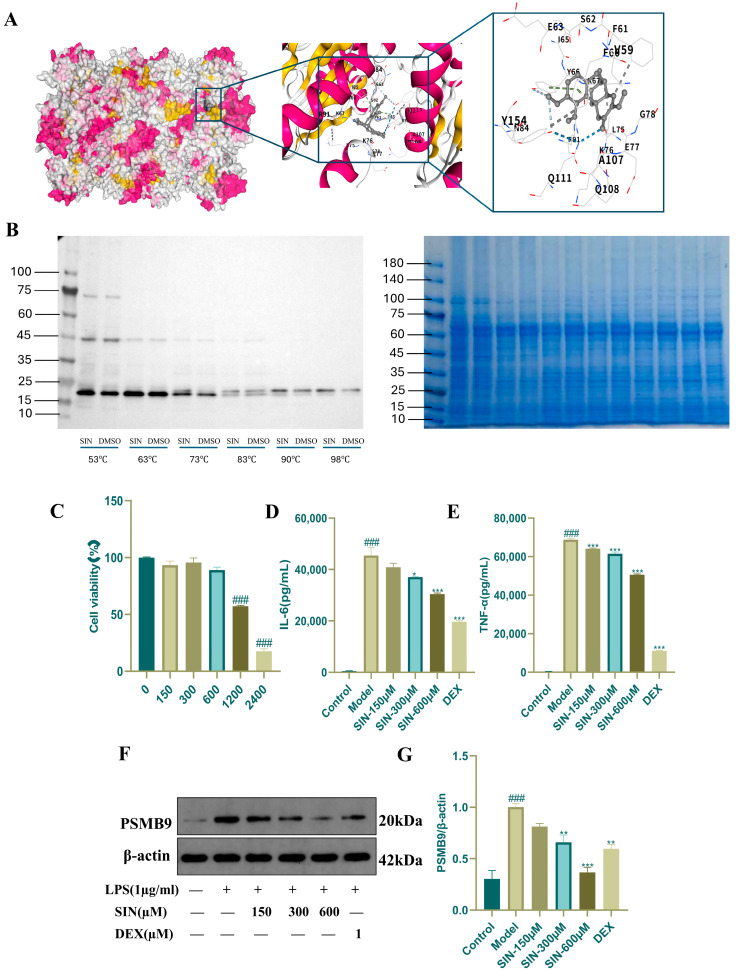
Molecular docking and cell experiments verified the regulatory effect of SIN on PSMB9 protein. (**A**) Molecular docking mode diagram of PSMB9 and SIN. (**B**) Cellular thermal shift assay (CETSA) showing the thermal stability of PSMB9 in the presence or absence of SIN. (**C**) Effect of SIN on RAW264.7 cell (macrophages) viability. (**D**) Effect of SIN on interleukin-6 (IL-6) expression in the supernatant of LPS-induced macrophages. (**E**) Effect of SIN on tumor necrosis factor-α (TNF-α) expression in different groups. (**F**) Western Blot band display of SIN pretreatment on PSMB9 protein in different groups. (**G**) Effect of SIN pretreatment on the expression of PSMB9 protein in different groups. Results were expressed as mean ± standard deviation (SD). (Note: Compared with the normal group, ^###^ *p* < 0.001; compared with the model group, *** *p* < 0.001, ** *p* < 0.01, * *p* < 0.05).

Molecular docking analysis revealed a Vina score of −9.0 kcal/mol for the PSMB9–SIN interaction, indicating robust binding affinity (Figure 9A). CETSA assay was performed and the result revealed that as the temperature increases, the PSMB9 band gradually weakens, indicating that SIN exert a weak stabilizing effect on the PSMB9 protein (Figure 9B).

The results of CCK-8 assay (Figure 9C) showed that when the concentration of SIN exceeded 600 μM, it may produce cytotoxic effects. Therefore, we selected the SIN concentration range (150–600 μM) without obvious cytotoxicity for subsequent experiments. ELISA results indicated that SIN dose-dependently inhibited the concentrations of IL-6 and TNF-α in the culture supernatant, a trend also observed with DEX (Figure 9D,E). Western blot analysis revealed that SIN downregulated PSMB9 protein levels in a dose-dependent manner. Similarly, DEX treatment also led to a significant inhibition of PSMB9 expression (Figure 9F,G). These results suggest that SIN exerts anti-inflammatory effects, at least in part, through downregulation of PSMB9 in LPS-induced macrophages.

## 4. Discussion

The dysregulation of immune responses involving multiple inflammatory signaling pathways is important in RA progression. Continuous crosstalk between infiltrating immune cells and FLS sustains synovial inflammation and progressive joint damage. The activation of immune-associated pathways, including JAK–STAT signaling, interferon responses, Toll-like receptor signaling, and NF-κB pathways, was found in integrative transcriptomic analyses across multiple independent cohorts, further supporting the concept of immune-driven transcriptional remodeling representing a defining feature of RA synovial pathology. Consistent with this, the RA group displayed increased infiltration of macrophages, T cells, and dendritic cells, highlighting the importance of FLS crosstalk in disease progression. Macrophages, especially M1 polarization macrophage, emerged as main contributors to the inflammatory milieu among these populations, which match with their established role as major producers of pro-inflammatory cytokines. These observations support a model in which persistent activation of innate immune programs reinforces chronic tissue inflammation and shapes the pathogenic microenvironment of RA.

The results of network-level analysis further suggested that RA-associated transcriptional changes are organized in coordinated gene programs, and PSMB9 emerged as a centrally connected gene together with TAP1 and CFB. Of note, the central positioning of PSMB9 in the network suggests that PSMB9 may act as a regulatory node linking antigen-processing machinery with inflammatory signaling and contribute to synchronized immune activation in RA synovium. Notably, PSMB9 dysregulation extended beyond infiltrating immune cells and was also evident in FLS, cells increasingly recognized as active drivers of RA pathology. Elevated PSMB9 expression in RA-derived FLS was associated with enhanced cytokine and interferon signaling, suggesting that inflammatory activation in RA is not solely immune-cell driven but may also arise from intrinsic alterations within resident stromal cells. These findings support a model in which immune and stromal compartments cooperatively sustain synovial inflammation, with PSMB9 functioning at the interface of these cellular systems.

Although SIN has long been used for the treatment of RA, the molecular basis underlying its anti-inflammatory effects has remained incompletely defined. Previous studies have linked PSMB9 expression to inflammatory activation and macrophage polarization. In this study, SIN treatment suppressed PSMB9 expression in macrophages. In addition, molecular docking predicted a potential interaction between SIN and PSMB9, and CETSA revealed a modest stabilizing effect of SIN on PSMB9, suggesting possible target engagement. In line with these findings, suppression of PSMB9 expression by SIN was accompanied by reduced production of IL-6 and TNF-α in macrophages, suggesting that modulation of PSMB9 represent one mechanism through which SIN attenuates inflammatory responses. These findings raise the possibility that targeting proteasome-associated immune regulators may represent an effective strategy for modulating pathological inflammation in RA. However, sinomenine likely acts through multiple mechanisms, including antioxidant pathways such as Nrf2. The interaction with PSMB9 should therefore be considered a potential, rather than definitive, mechanism.

However, several limitations of this study should be acknowledged. First, functional validation of the SIN-PSMB9 interaction and its anti-inflammatory effects was performed exclusively in RAW264.7 macrophages, a murine macrophage cell line stimulated with LPS. Although this model is widely used for mechanistic studies of macrophage inflammation, it represents a simplified inflammatory paradigm that does not adequately recapitulate the complex cellular heterogeneity and chronic autoimmune microenvironment of human RA synovium. Secondly, we did not perform in vivo validation in this study. Finally, functional experiments in this research mainly relied on a single cell line instead of multiple cell lines, which may not fully capture the cellular heterogeneity of human synovial tissue. Future studies incorporating animal models and primary patient-derived cells will be required to define the mechanistic role of PSMB9 further and evaluate its therapeutic potential.

Overall, our findings suggest PSMB9 as a molecular bridge connecting immune activation and stromal dysfunction in RA synovium and also provide mechanistic insight into the action of SIN and support PSMB9 as a candidate target for therapeutic modulation of inflammatory pathways. Notably, the single-cell analysis in this study is based on a limited number of donors and should be considered exploratory, and the diagnostic potential of PSMB9 requires further validation in larger cohorts and independent datasets.

## 5. Conclusions

In summary, PSMB9 is a key immune-associated gene in RA, linked to macrophage infiltration and inflammatory activation. SIN may partially exert its anti-inflammatory effects through modulation of PSMB9, although further validation is required.

## Data Availability

The original data presented in the study are openly available in the Gene Expression Omnibus (GEO) database under accession numbers GSE276772, GSE285977, GSE55457, and GSE77298. Additional experimental data are available from the corresponding author upon reasonable request due to institutional regulations regarding data management and storage.

## Abbreviations

RA	rheumatoid arthritis
NSAIDs	non-steroidal anti-inflammatory drugs
DMARDs	disease-modifying anti-rheumatic drugs
SIN	sinomenine
MTX	methotrexate
PSMB9	proteasome subunit beta type-9
LMP2	low molecular mass protein 2
IFN-γ	interferon-γ
WGCNA	weighted gene co-expression network analysis
RMA	robust multi-array average
GO	Gene Ontology
KEGG	Kyoto Encyclopedia of Genes and Genomes
BP	biological process
CC	cellular component
MF	molecular function
GSEA	gene set enrichment analysis
MSigDB	Molecular Signatures Database
PPI	protein–protein interaction
OA	osteoarthritis
CPM	counts-per-million
LPS	lipopolysaccharide
DEX	dexamethasone
TOM	topological overlap matrix
FLS	fibroblast-like synoviocytes
CFB	Complement Factor B
